# A Novel Classification of Glioma Subgroup, Which Is Highly Correlated With the Clinical Characteristics and Tumor Tissue Characteristics, Based on the Expression Levels of Gβ and Gγ Genes

**DOI:** 10.3389/fonc.2021.685823

**Published:** 2021-06-18

**Authors:** Zehao Cai, Chunna Yu, Shenglan Li, Can Wang, Yaqiong Fan, Qiang Ji, Feng Chen, Wenbin Li

**Affiliations:** Department of Neuro-oncology, Cancer Center, Beijing Tiantan Hospital, Capital Medical Unversity, Beijing, China

**Keywords:** glioma, G protein subunit, RNA sequencing data, subgroup classification, prognosis

## Abstract

**Purpose:**

Glioma is a classical type of primary brain tumors that is most common seen in adults, and its high heterogeneity used to be a reference standard for subgroup classification. Glioma has been diagnosed based on histopathology, grade, and molecular markers including IDH mutation, chromosome 1p/19q loss, and H3K27M mutation. This subgroup classification cannot fully meet the current needs of clinicians and researchers. We, therefore, present a new subgroup classification for glioma based on the expression levels of Gβ and Gγ genes to complement studies on glioma and Gβγ subunits, and to support clinicians to assess a patient’s tumor status.

**Methods:**

Glioma samples retrieved from the CGGA database and the TCGA database. We clustered the gliomas into different groups by using expression values of Gβ and Gγ genes extracted from RNA sequencing data. The Kaplan–Meier method with a two-sided log-rank test was adopted to compare the OS of the patients between GNB2 group and non-GNB2 group. Univariate Cox regression analysis was referred to in order to investigate the prognostic role of each Gβ and Gγ genes. KEGG and ssGSEA analysis were applied to identify highly activated pathways. The “estimate” package, “GSVA” package, and the online analytical tools CIBERSORTx were employed to evaluate immune cell infiltration in glioma samples.

**Results:**

Three subgroups were identified. Each subgroup had its own specific pathway activation pattern and other biological characteristics. High M2 cell infiltration was observed in the GNB2 subgroup. Different subgroups displayed different sensitivities to chemotherapeutics. GNB2 subgroup predicted poor survival in patients with gliomas, especially in patients with LGG with mutation IDH and non-codeleted 1p19q.

**Conclusion:**

The subgroup classification we proposed has great application value. It can be used to select chemotherapeutic drugs and the prognosis of patients with target gliomas. The unique relationships between subgroups and tumor-related pathways are worthy of further investigation to identify therapeutic Gβγ heterodimer targets.

## Introduction

Glioma is a classical type of primary brain tumors that is most common seen in adults, and its high heterogeneity used to be a primitive feature for subgroup classification (1). Historically, glioma was diagnosed based on histopathology and grade (2). World Health Organization Classification of Tumors of the Central Nervous System, revised in 2016, added several molecular markers, including IDH mutation, chromosome 1p/19q loss, and H3K27M mutation into an integrated glioma diagnosis (3). With the rise of genomic medicine, this paper, proposing a signature with multiple genes as the indicator of subgroup classification, has adopted an increasingly usual method. A research group described a gene expression-based molecular classification of GBM into Proneural, Neural, Classical, and Mesenchymal subtypes (4). Some studies designed signatures with multiple genes related to m6A RNA methylation, ferroptosis, and lipid metabolism to stratify the prognosis of gliomas (5–7). The effect of certain biological processes on gliomas lied in the focus of the above studies. Based on the observation to the expression levels of Gβ and Gγ genes, we found that they had the potential to be molecular markers in subgroup classification of glioma.

G protein-coupled receptors, the largest family of cell-surface receptors in the human genome, are capable of mediating the signaling of a wide range of ligands, hormones, neurotransmitters, proteases, lipids, and peptides, for instance (8). GPCR activation is mediated by the binding of the GPCR extracellular domain with the agonist ligand. GDP on the Gα subunit is replaced by GTP, resulting in the dissociation of the Gα subunit from the Gβγ heterodimer. Gβγ heterodimer reacts on Phospholipase C, Voltage-Dependent Ca2^+^ Channels, Phosphoinositide 3 Kinases, Mitogen-Activated Protein Kinases, and is also involved in microtubule polymerization, recycling endosomes, and Golgi fragmentation (9–15). Furthermore, Gβ and Gγ may be involved in the assembly of particular GPCR complexes. The pool of Gβ and Gγ in a particular cell may drive and/or dictate which GPCR complexes can form in that cell (16). Gβ and Gγ are crucial participants in the malignant progression of tumors. GNB4 overexpression activates the Erk1/2 pathway resulting the process of epithelial-mesenchymal transformation of GC (17). The proliferation of SK-Mel28 human malignant melanoma cells was suppressed with overexpressed GNG2, and the mean tumor size of overexpressed-GNG2 SK-Mel28 cells was less than that of the controlled SK-Mel28 cells in nude mice after inoculation (18).

There are five β-subunits (β1, β2, β3, β4, β5) and 12 γ-subunits (γ1, γ2, γ3, γ4, γ5, γ7, γ8, γ9, γ10, γ11, γ12, γ13) in the human body. βγ pairs are specifically related to downstream signals (19). Gβ1γ2 heterodimer activates PI3K, whereas Gβ5γ2 heterodimer does not possess the similar effect. Both of the above two heterodimers can activate PLCb1 and PLCb2, yet only Gβ1γ2 is able to activate PLCb3 (20). Differences in affinities between several types of G protein subunits will impose restrictions on the formation of certain heterotrimers and, on the other hand, determine the activity of certain type of G protein in a cell (21). Gγ2 and Gγ3 are more likely to be bound to Gβ1, Gβ2, and Gβ4 subunits, whereas Gβ2 is not bound to Gγ1, Gγ11, Gγ13, and is only weakly bound to Gγ8 (22, 23). The mutation rate of Gβ and Gγ genes in glioma remains low, so the influence of mutations can be ignored in the subgroup classification.

## Methods

### Patients and Datasets

Nine hundred fifty-one glioma samples retrieved from the CGGA database (http://www.cgga.org.cn) and 672 glioma samples retrieved from TCGA database (http://cancergenome.nih.gov/) were utilized in this study for reference. Relevant data included relapse samples. For the same patient, we only used the first tumor RNA sequencing data. The FPKM-standardized mRNA sequencing data was log2 transformed for all analyses. The count format mRNA sequencing data retrieved from TCGA was standardized by voom function.

### Bioinformatic Analysis

We firstly extracted the expression values of Gβ and Gγ genes from mRNA sequencing data. Then, we clustered the gliomas into different groups with “Consensus Cluster Plus” package for R v4.0.3 (https://www.r-project.org/). PCA was employed to study the gene expression patterns in different glioma groups. We applied the first three PC values representing RNA sequencing data of each sample to establish a distribution map of the sample. Drug sensitivity analysis was later on performed with “pRRophetic” package (24, 25), resulting in a lower IC50. And this indicated that the subgroup was more sensitive to the drug. Then, we screened out differentially expressed genes between each two subgroups with “DESeq2” package (26). The DEG threshold was set at a |log2 fold change| ≥ 1 and an adjusted P value <0.05. KEGG pathway enrichment analysis was used to annotate DEG. The reliability of the results was verified using ssGSEA. Gene lists of pathways used in ssGSEA were downloaded from the KEGG website (https://www.kegg.jp/kegg/pathway.html). The “estimate” package, “GSVA” package, and the online analytical tools CIBERSORTx (https://cibersortx.stanford.edu/) were employed to evaluate immune cell infiltration in glioma samples (27).

### Statistical Analysis

Student’s t-tests performed in SPSS v26 were used to determine the differences of Gβ and Gγ genes expression level. When the expression level of a gene in a subgroup was significantly higher than that in the other two groups, it was considered that the gene was specifically highly expressed in this subgroup and *vice versa*. Chi-square tests were used to compare the distribution of clinical features between three groups. The Kaplan–Meier method with a two-sided log-rank test was referred to compare the OS of the patients between GNB2 group and non-GNB2 group. Univariate Cox regression analysis on the expression levels in CGGA and TCGA dataset was used to investigate the prognostic role of each Gβ and Gγ gene. Pearson method was used to evaluate the correlation between Gβ and Gγ genes and macrophage infiltration. On one hand, a R value more than 0.5 was considered a significant positive correlation. On the other hand, a p value less than 0.05 was considered to be statistically significant.

## Results

### Three Types of Gβγ-Related Subgroups Existing in Glioma

**Figure 1:**
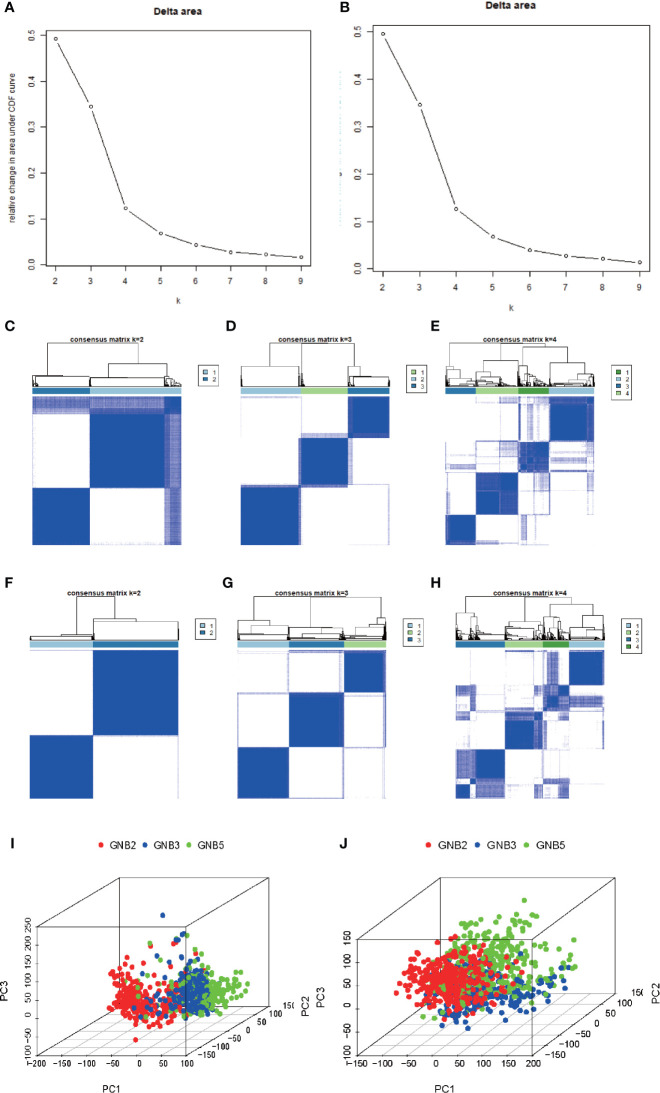
Selection of K value of subgroup classification. Relative change in area under CDF curve for k = 2 to 9 in the TCGA dataset **(A)** and the CGGA dataset **(B)**. The correlation of samples between subgroups in the TCGA dataset **(C–E)** and the CGGA dataset **(F–H)**. At k = 3, the whole gene expression pattern of gliomas performed using Principal Component Analysis in the TCGA dataset **(I)** and CGGA dataset **(J)**.

**Figure 2:**
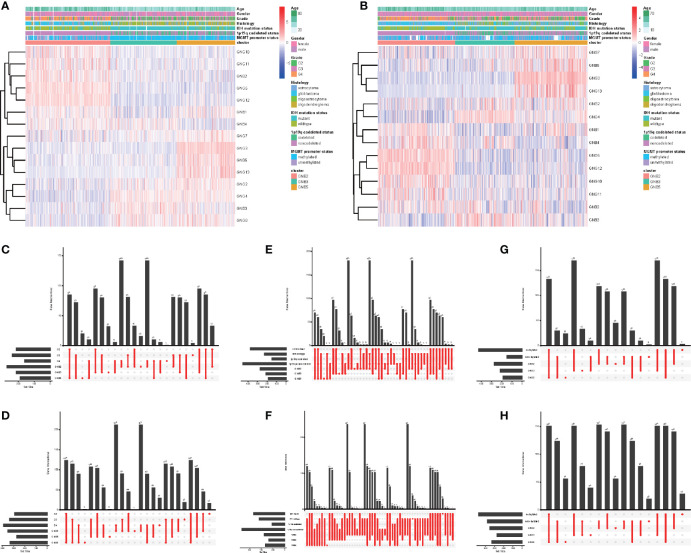
Subgroups in gliomas with different clinicopathological features. The correlation between expression levels of Gβ and Gγ genes in gliomas with subgroups and clinicopathological features in the TCGA dataset **(A)** and the CGGA dataset **(B)**. Number of patients in each subgroup with grade in the TCGA dataset **(C)** and the CGGA dataset **(D)**. Number of patients in each subgroup with IDH mutant status and 1p19q codeleted status in the TCGA dataset **(E)** and the CGGA dataset **(F)**. Number of patients in each subgroup with MGMT methylated status and MGMT methylated status in the TCGA dataset **(G)** and the CGGA dataset **(H)**.

Based on the clustering consistency (**Figures 1A, B**, note that an inflection point appeared at k = 4, indicating that 4 was the best value) and the correlation of samples between subgroups (**Figures 1C–H**, note that there was a high correlation of samples between subgroups at k = 4, which was significantly improved at k = 3) between two datasets, k = 3 seemed to be the sound selection (**Figures 1I, J**), we found that the subgroups of the two datasets matched in accordance (**Figures 2A, B**). GNB2, GNB5, GNG10, GNG11, and GNG12 were highly expressed, while GNB3, GNG2, GNG4, and GNG13 were low expressed in a subgroup that we named “GNB2 subgroup.” GNB3 was highly expressed while GNB1 and GNG12 were low expressed in a subgroup named “GNB3 subgroup.” GNB5, GNG3, GNG7, and GNG13 were highly expressed, while GNB2 and GNB4 were low expressed in a subgroup named “GNB5 subgroup.”

### Significant Differences Demonstrated in Molecular and Clinical Characteristics Between Different Subgroups

What was particularly notable was that the GNB2 subgroup was almost entirely composed of non-1p19q codeletions in TCGA (99.2%) and CGGA (98.6%) datasets (**Figures 2E, F**). This was because of the position of GNG5 and GNG12, that were highly expressed in GNB2 subgroup, both of which were located at the position of chromosome 1p. In addition, GNB1 was located on chromosome 1p and GNG8 on chromosome 19q. There was no significant difference in codeleted 1p19q rate between the GNB3 subgroup and the GNB5 subgroup (**Figures 2E, F**). GNB2 subgroup was also associated with higher rates of high pathological grade (**Figures 2C, D**), wild type IDH (**Figures 2E, F**) and unmethylated MGMT promoter (**Figures 2G, H**). While GNB3 subgroup was associated with higher rate of mutated IDH and methylated MGMT promoter (**Figures 2G, H**). There was no sufficient evidence to show a significant relationship between subgroups and tumor location.

**Figure 3:**
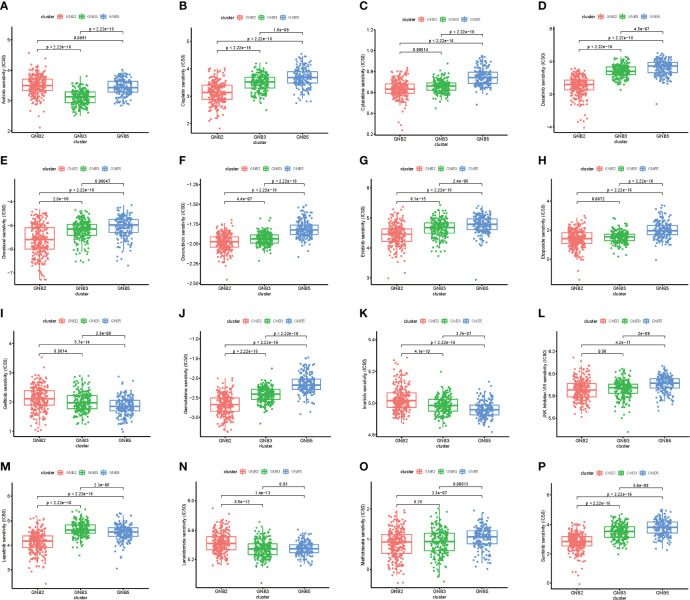
The sensitivity to chemotherapy in three subgroups. Estimated IC50 of chemotherapeutic drugs including Axitinib **(A)**, Cisplatin **(B)**, Cytarabine **(C)**, Dasatinib **(D)**, Docetaxe **(E)**, Doxorubicin **(F)**, Erlotinib **(G)**, Etoposide **(H)**, Gefitinib **(I)**, Gemcitab **(J)**, Imatinib **(K)**, JNK.Inhibitor.VIII **(L)**, Lapatinib **(M)**, Lenalidomide **(N)**, Methotrexate **(O)**, Sunitinib **(P)**.

We then investigated the response to chemotherapy in three subgroups before we arrived at the conclusion that 16 chemotherapeutic drugs displayed significant differences in estimated IC50 between three subgroups (**Figure 3**). Patients in GNB2 subgroup showed the highest sensitivity to 11 chemotherapies, including cisplatin (**Figure 3B**), cytarabine (**Figure 3C**), and etoposide (**Figure 3H**), which was consistent with the result that subgroup with higher malignancy was more sensitive to chemotherapies (28). In contrast, patients in GNB5 subgroup showed the lowest sensitivity to 11 chemotherapies. There was no significant difference between GNB2 subgroup and GNB3 subgroup in the sensitivity to methotrexate, which was used for CSF injection in glioma patients with spinal dissemination, and both were higher than that in GNB5 subgroup (**Figure 3O**).

### Significant Biological Differences Among Subgroups

**Figure 4:**
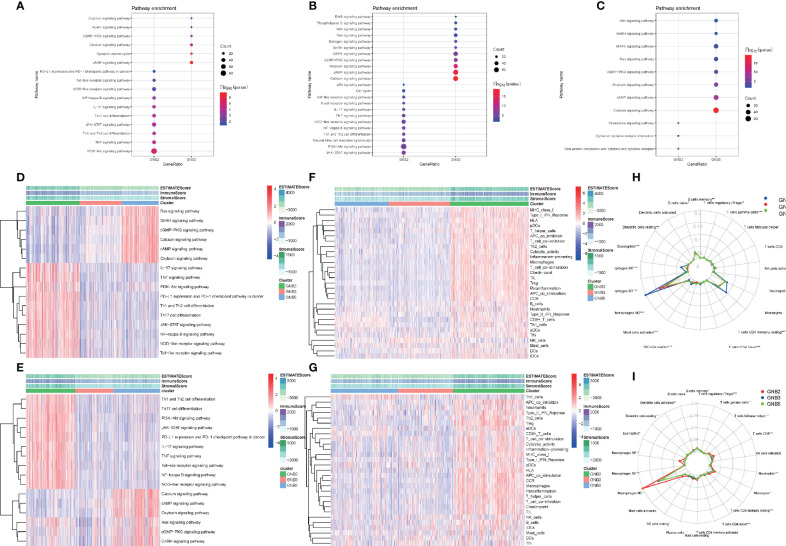
Biological differences among different subgroups of gliomas. Pathways that genes upregulated in GNB2 subgroup and GNB3 subgroup enriched in **(A)**. Pathways that genes upregulated in GNB2 subgroup and GNB5 subgroup enriched in **(B)**. Pathways that genes upregulated in GNB3 subgroup and GNB5 subgroup enriched in **(C)**. Correlation between subgroups and selected pathways activation in the TCGA dataset **(D)** and the CGGA dataset **(E)**. GNB2 subgroup was associated with strong stemness and immune inflammation in the TCGA dataset **(F)** and the CGGA dataset **(G)**. Immune cells infiltrating gliomas in the TCGA dataset **(H)** and the CGGA dataset **(I)**.

We screened for differentially expressed genes between each of the two subgroups in TCGA dataset, and KEGG pathway analysis were carried out to understand which pathways the up-regulated and down-regulated genes were enriched in (**Figures 4A-C**). Sixteen tumor-related pathways with strong stability were selected for further ssGSEA analysis in TCGA (**Figure 4D**) and CGGA (**Figure 4E**) datasets. The results concluded from TCGA and CGGA datasets showed strong consistency. GNB2 subgroup was highly associated with high activation of PI3K−Akt signaling pathway, JAK−STAT signaling pathway, and several immune-related pathways. As for GNB5 subgroup, it was highly associated with high activation of Calcium signaling pathway, GnRH signaling pathway, Ras signaling pathway, and other pathways. Last but not the least, GNB3 subgroup was not associated with activation of the 16 selected pathways.

Considering the relationship between GNB2 subgroup and immune-related pathways, we evaluated immune infiltration with ESTIMATE algorithm and ssGSEA of 29 immune-related gene sets. The results indicated that the GNB2 subgroup was associated with strong stemness and immune inflammation (**Figures 4F, G**). When characterizing the abundances of different immune cell types with CIBERSORTx, we found that the infiltration levels of M0 macrophages and M2 macrophages increased significantly in glioma samples of GNB2 subgroup in both the CGGA (**Figure 4H**) and TCGA (**Figure 4I**) datasets. In gliomas, tumor-associated macrophages were promoted by glioma-secreted cytokines to acquire M1 or M2 phenotype, which differs in relation to microenvironment modulation (29, 30). On the purpose of further exploring the association between core genes of GNB2 subgroup and macrophages, characteristic markers of TAMs, M1, and M2 were selected to perform Pearson analysis (31, 32). Relevant results showed that GNG5 and GNG12 were positively correlated with TAMs and M2, yet not with M1 (**Figures S1A, B**). Results of patients with non-codeleted 1p19q displayed a significant reduction of the correlation between GNG12 and M2, but this did not occur concerning to GNG5 (**Figures S1C, D**). The result revealed the correlation between GNG12 and M2 macrophages, which was not dependent on the increased expression level of GNG12 expressed by macrophages resulted by the increase in the number of M2 macrophages, but on the involvement of GNG12 expressed by glioma cells in M2 macrophages infiltration. Through the analysis of publicly available single-cell RNA sequencing data, we found that cells with high GNG12 expression were mainly glioma cells, which supported this conclusion (**Figures S1E, F**).

### Poor Survival in Patients With Gliomas Predicted by GNB2 Subgroup

**Figure 5:**
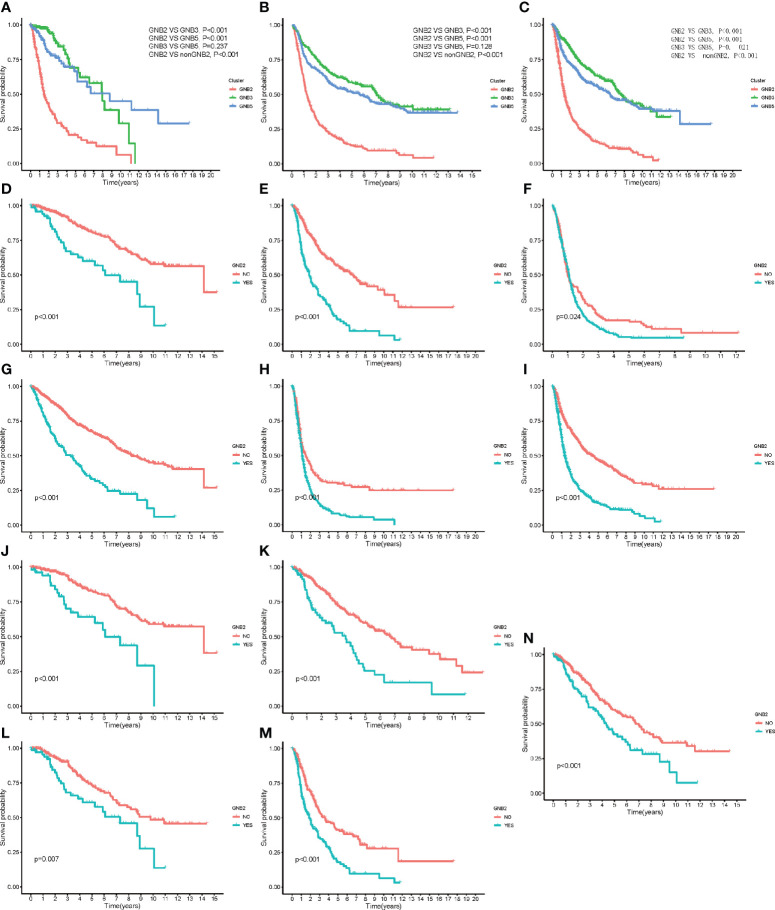
Kaplan–Meier overall survival curves for subgroups. Kaplan–Meier overall survival curves for patients in the TCGA dataset **(A)** and CGGA dataset **(B)**. OS curves for patients in the merged dataset **(C)**. OS curves for patients with grade 2 **(D)**, grade 3 **(E)**, and grade 4 **(F)**. OS curves for patients with mutated IDH **(G)**. OS curves for patients with wildtype IDH **(H)**. OS curves for patients with non-codeleted 1p19q **(I)**. OS curves for patients with mutated IDH with grade 2 **(J)** and grade 3 **(K)**. OS curves for patients with non-codeleted 1p19q with grade 2 **(L)** and grade 3 **(M)**. OS curves for LGG patients with mutated IDH with non-codeleted 1p19q **(N)**.

The characteristics of GNB2 subgroup, including IDH wildtype, 1p19q non-codeletion, high infiltration of M2 macrophages, all predicted poor survival in patients with gliomas. Consequently, we then conducted the Kaplan-Meier survival analysis. Significant correlation was observed between GNB2 subgroup and decreased OS was observed in patients with glioma as well (**Figures 5A, B**). After combining cluster and survival information of two datasets, we found that patients in GNB3 subgroup had longer OS than those in GNB5 subgroup (**Figure 5C**). Based on the reduction of sample size and data compatibility, we applied the combined data to the subgroup analysis. In patients with grade 2 (**Figure 5D**), grade 3 (**Figure 5E**), grade 4 (**Figure 5F**), we all observed a significantly shorter OS in the GNB2 subgroup than the non-GNB2 subgroup. GNB2 subgroup also exhibited worse OS in patients with glioma with mutated IDH (**Figure 5G**), wild type IDH (**Figure 5H**), and non-codeleted 1p19q (**Figure 5I**). In both patients with mutated IDH and patients with non-codeleted 1p19q, patients with grade 2 and grade 3 in GNB2 subgroup showed shorter OS than those in non-GNB2 subgroup (**Figures 5J–M**). In patients with LGG with mutated IDH and non-codeleted 1p19q, a finely segmented patient set, GNB2 subgroup predicted poor survival, too (**Figure 5N**). This result was encouraging because there were no further officially recommended prognostic molecular markers available for these patients with LGG with mutation IDH and non-codeleted 1p19q.

We afterwards performed a univariate Cox regression analysis on the expression levels in TCGA (**Figure S2A**) and CGGA (**Figure S2B**) dataset outing to investigate the prognostic role of each Gβ and Gγ gene. The results showed that high GNB1, GNB2, GNG5, GNG10, GNG11, GNG12 expression were associated to poor prognosis and GNB5, GNG4 were associated to good prognosis in both TCGA and CGGA datasets.

## Discussion

We also referred to RNA sequencing data from other tumors, including LUAD and LUSC, for cluster analysis. Relevant results, reflecting strong correlations and a lack of valuable pathways, were unsatisfactory, though. The positive clustering results of this study might be attributed to some characteristics of glioma tissue, such as the glioma-specific 1p19q co-deletion that affected the expression of GNB1, GNG5, GNG7, GNGN8, and GNG12. Besides, compared to other somatic tumors, the relatively immune-privileged microenvironment of glioma, which was dominated by macrophages, reduced the confiding effect of gene expression of other immune cells on the RNA sequencing data of the whole tissue.

Peripheral blood derived macrophages and intracranial microglia replaced T cells as the crucial immune cells in the immune microenvironment of glioma thanks to the existence of the blood brain barrier (33, 34). In the microenvironment of malignant tumors, M2 macrophages were the major subtype of macrophages and the important contributors to an immunosuppressive phenotype (35, 36). High M2 macrophages infiltration was associated with poor prognosis in patients with glioma, which partly explained the short OS in GNB2 patients. GNG12 might play a distinct role in the formation of immunosuppressive phenotype of glioma. A previous study showed that GNG12 did regulate PD-L1 expression by activating NF-κB signaling in pancreatic ductal adenocarcinoma. In this study, the expression level of GNG12 was also positively correlated with the expression of PD-L1.

Several molecular markers of GNB2 subgroup were associated with tumor progression. Both mutation and overexpression of GNB2 caused leukemogenesis, let alone downregulation of GNB2 expression reduced proliferative potential of tumor cells (37). Overexpression of GNG5 was associated with pool prognosis in patients with glioma (38). GNG4 was found to be one of the most hyper methylated and down regulated genes in GBM, and exogenous over expression of GNG4 inhibited SDF1α/CXCR4-dependent chemokine signaling leading to inhibition of proliferation and colony formation of GBM cell lines (39). High rates of high pathological grade and IDH wildtype were also the reasons for the poor prognosis of patients in GNB2 subgroup.

The limitation to our study is as follows. Due to the increasing complexity of subunit pairs, we did not incorporate Gα gene in this study. In addition, we did not obtain specific pairs of Gβ and Gγ in the corresponding subgroups that were difficult to get from the analysis of RNA sequencing data. A large number of experiments are still in need to determine exact pairs, despite the specificity of the combination of Gβ and Gγ is beneficial to narrow the scope. On the other hand, validating the subgroup model’s predictive capability on independently generated data does make a difference. Besides, this classification was obtained by unsupervised consistent clustering, which failed to presuppose specific conditions of G protein subunit gene expression value. To determine which subgroup a glioma tissue belongs to, we need the exact condition of each gene expression value or a mathematical determination model such as neural network model, which needed a certain number of samples would for parameter optimization. The RNA sequencing data we analyzed sourced from TCGA and CGGA databases, which limited the access to clinical data, such as extent of surgical resection and volume of the residue of tumor. A new clinical cohort collecting substantial clinical data for verification and further study is necessary. We identified several important pathways corresponding to subgroups, yet the role of Gβγ in these pathways and relevant effects of these pathways on tumor tissue remain to be further investigated.

## Conclusion

This paper has presented a new subgroup classification for glioma based on the expression level of Gβ and Gγ genes. Patients with glioma were divided into three subgroups that differed significantly from each other. Each subgroup has its own specific pathway activation pattern and other biological characteristics. The unique relationships between subgroups and tumor-related pathways can be further investigated to identify therapeutic Gβγ heterodimer targets. High M2 cell infiltration was observed in GNB2 subgroup. And GNG12 could be treated as a potential effector in immunosuppressive phenotype of glioma. Different subgroups have different sensitivities to chemotherapeutics, so this study may be referred to for clinical drug selection. Additionally, GNB2 subgroup predicted poor survival in patients with gliomas, especially in patients with LGG with mutation IDH and non-codeleted 1p19q. This subgroup classification is expected to be a new molecular marker to predict the prognosis of these patients. This classification can be used to screen out the patients with high actual malignant tumor in patients with low pathological grade, so as to recommend optimal treatment time in advance and to improve the possibility of treatment.

## Data Availability Statement

Publicly available datasets were analyzed in this study. This data can be found here: (http://www.cgga.org.cn, http://cancergenome.nih.gov/).

## Ethics Statement

The studies involving human participants were reviewed and approved by the ethics committee of Beijing Tiantan Hospital. Written informed consent for participation was not required for this study in accordance with the national legislation and the institutional requirements.

## Author Contributions

ZC is responsible for data analysis and article writing. WL is responsible for topic selection and research design. CY and YF are responsible for assisting in writing the paper. CW and QJ are responsible for guiding the statistical analysis. SL is responsible for assisting in writing the paper. FC directing the paper writing. All authors contributed to the article and approved the submitted version.

## Funding

This research was funded by the National Natural Science Foundation of China, grant number 81972338. This study was also financially funded by Beijing Municipal Health Commission of China, Advanced Research and Training Program of Beijing Double Leading Scholars from China Academy of Chinese Medical Science and National Science and Technology Major Project of China (No. 2016ZX09101017).

## Conflict of Interest

The authors declare that the research was conducted in the absence of any commercial or financial relationships that could be construed as a potential conflict of interest.

## Supplementary Material

Supplementary Figure 1 The correlation between core genes expression level of GNB2 subgroup and M2 macrophages infiltration. Correlation between core genes expression level of GNB2 subgroup and infiltration of M0, M1, and M2 macrophages in GNB2 subgroup patients in the TCGA dataset **(A)** and the CGGA dataset **(B)**. Correlation between core genes expression level of GNB2 subgroup and infiltration of M0, M1, and M2 macrophages in patients with codeleted 1p19q in the TCGA dataset **(C)** and the CGGA dataset **(D)**. Cell types from each cluster analyzed from single-cell sequencing data **(E)**. The expression level of GNG12 gene in each cluster **(F)**.

Supplementary Figure 2 Univariable Cox regression analysis of Gβ and Gγ genes in the TCGA dataset **(A)** and the CGGA dataset **(B)**.

## Abbreviations

GNG, Guanine Nucleotide Binding Protein, Gamma; GNB, Guanine Nucleotide Binding Protein, Beta; GBM, glioblastoma; LGG, low grade glioma; TCGA, The Cancer Genome Atlas; CGGA, Chinese Glioma Genome Atlas; CGP, Cancer Genome Project; PCA, Principal Component Analysis; PC, Principal Component; PD-L1, Programmed Cell Death 1 Ligand 1; KEGG, Kyoto Encyclopedia of Genes and Genomes; GSEA, Gene Set Enrichment Analysis; IDH, Isocitrate Dehydrogenase; MGMT, O-6-Methylguanine-DNA Methyltransferase; CSF, Cerebrospinal Fluid; MAPK, Mitogen-activated Protein Kinase; AMP, Adenosine Monophosphate; cAMP, Cyclic Adenosine Monophosphate; PLC, Phospholipase C; NF−κB, Nuclear Factor Kappa B Subunit; GDP, Guanosine diphosphate; DEG, Differentially expressed genes; GTP, Guanosine triphosphate; IC50, Half maximal inhibitory concentration.
