# Supplementary figures and images for: A Novel Classification of Glioma Subgroup, Which Is Highly Correlated With the Clinical Characteristics and Tumor Tissue Characteristics, Based on the Expression Levels of Gβ and Gγ Genes
Source: Front Oncol. 2021 Jun 18;11:685823. doi: 10.3389/fonc.2021.685823 (PMC8250418; doi:10.3389/fonc.2021.685823)

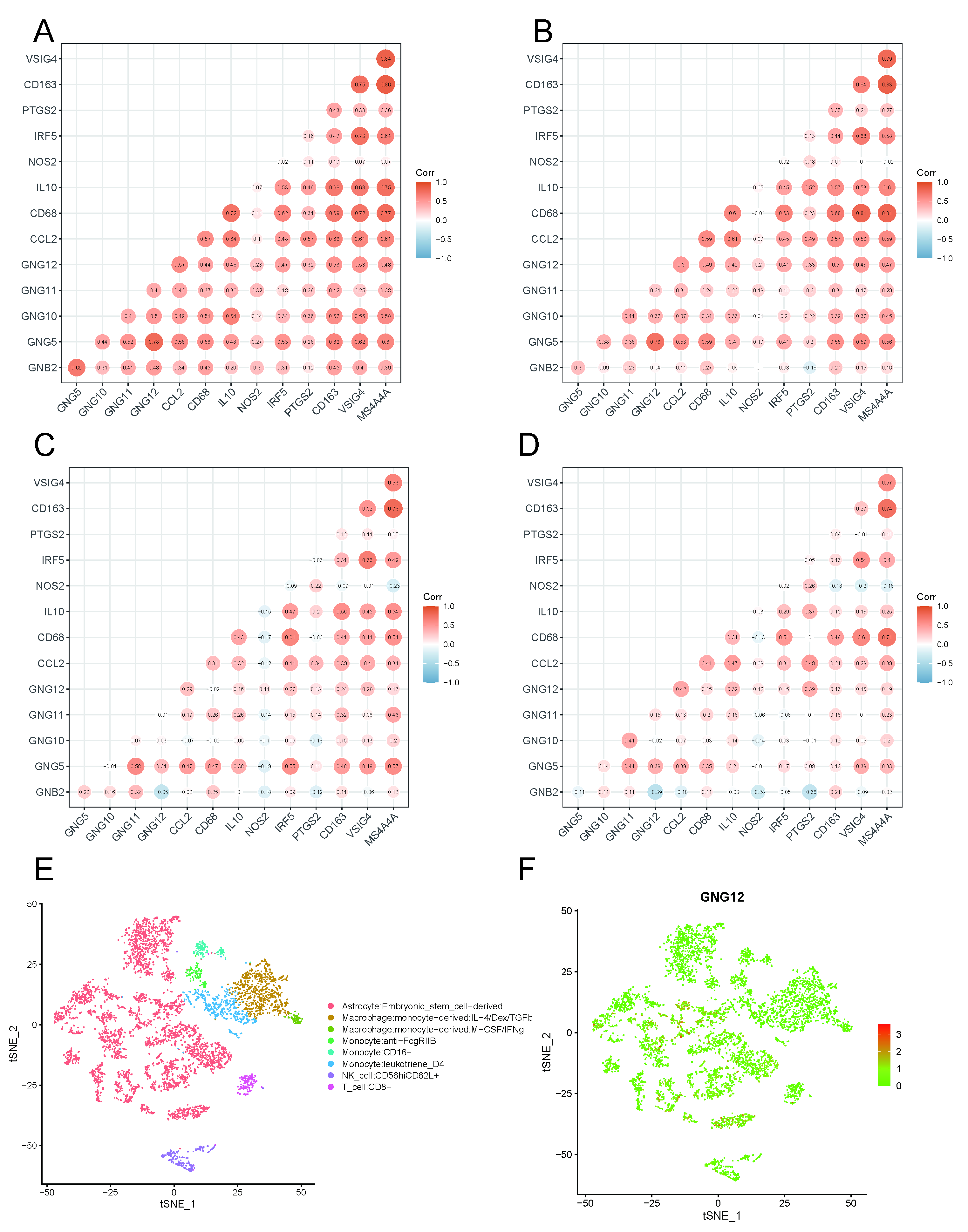

Supplement: Supplementary Figure 1 — The correlation between core genes expression level of GNB2 subgroup and M2 macrophages infiltration. Correlation between core genes expression level of GNB2 subgroup and infiltration of M0, M1, and M2 macrophages in GNB2 subgroup patients in the TCGA dataset (A) and the CGGA dataset (B). Correlation between core genes expression level of GNB2 subgroup and infiltration of M0, M1, and M2 macrophages in patients with codeleted 1p19q in the TCGA dataset (C) and the CGGA dataset (D). Cell types from each cluster analyzed from single-cell sequencing data (E). The expression level of GNG12 gene in each cluster (F). [file Image_1.tif]

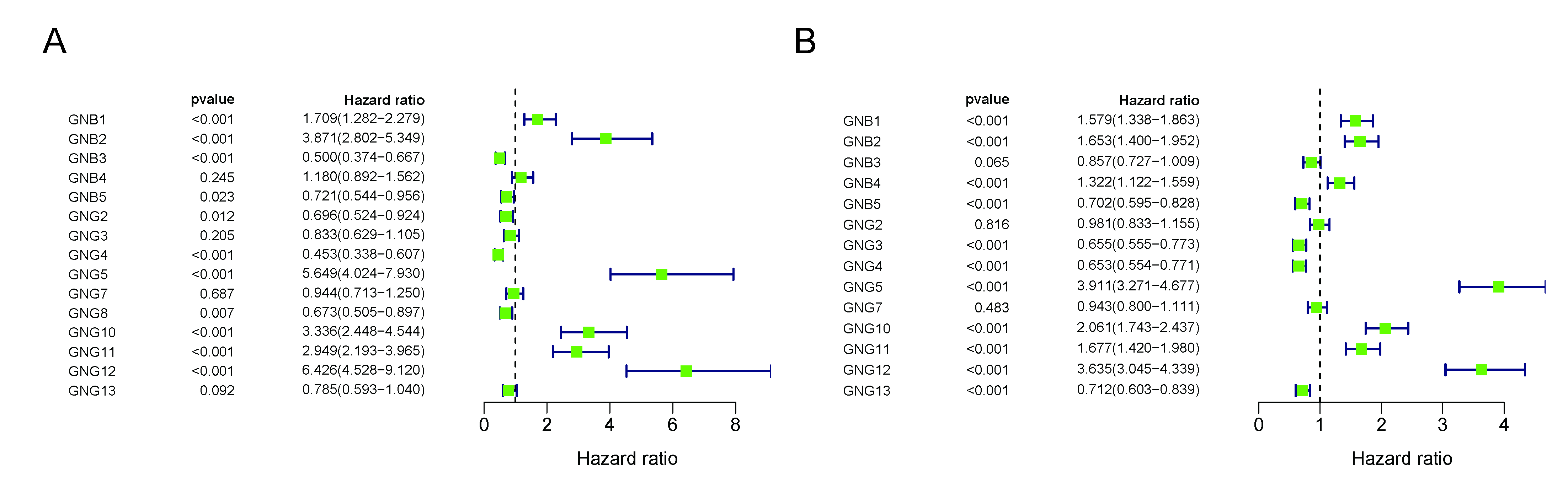

Supplement: Supplementary Figure 2 — Univariable Cox regression analysis of Gβ and Gγ genes in the TCGA dataset (A) and the CGGA dataset (B). [file Image_2.tif]
